# Pathological Conditions of the Urine

**Published:** 1904-06-25

**Authors:** 


### PATHOLOGICAL CONDITIONS OF THE URINE.

**Urology.**—*Hæmatoporphyrin*, when present in large amounts, is often due to taking sulphonal, but A. E. Garrod [1] mentions 12 cases where that cause did not exist. The majority of the patients were males, and in some the condition existed for years. The diseases from which they suffered were most various, and he does not think the condition is caused by excessive hæmolysis. The common excretion of slight amounts appears to depend on hepatic changes, but there is no conclusive evidence that these severe attacks are produced in the same way. *Black Urine.*—It is difficult to draw a line, says Garrod,[2] between urine which is black when passed, and that which becomes so on standing. Blackness may be due to (1) Jaundice, especially when of long standing, and when much biliverdin is excreted. (2) Hæmaturia, which can be detected by the spectroscope. (3) Hæmoglobinuria, as in the paroxysmal form and in "blackwater fever," and in poisoning by chlorate of potash and arseniuretted hydrogen. (4) Hæmatoporphyrinuria, where the colour is largely due to other unknown pigments. (5) Melanuria, due to melanotic sarcoma, when the viscera and especially the liver have been involved. The urine is generally normal when passed, but on exposure or on addition of nitric acid it becomes black. Ferric chloride also turns it black with a grey precipitate. (6) Alkaptonuria, a rare congenital condition. The urine rapidly becomes black if an alkali is added, and on merely standing in the air it does so slowly. It stains linen fabrics, and reduces Fehling's solution. (7) Ochronosis, or blackening of the cartilages, with or without pigmentation of the skin, which is another very rare state in which urine becomes black on standing. (8) Indican, which is sometimes in such excess as to cause blackening. The colour is not produced by cold nitric acid or ferric chloride, nor does it reduce Fehling. After adding HCl. and a drop of $HNO_3$, and heating, the pigment can be extracted by shaking up with chloroform. (9) In some cases of phthisis the urine becomes black if it is kept for a month or more. (10) It is said that some fruits, as well as resorcin, carbolic acid, and similar drugs cause dark urine, and large doses of rhubarb or senna may turn alkaline urine to a purple colour. Osler [3] reports two cases in which ochronosis appeared together with alkaptonuria. The patients were brothers, and both had been rejected by insurance offices for diabetes before the reducing agent was shown to be alkapton. The sclerotics, the cartilage of the external ears, and a patch of skin over the nose and cheeks were stained of a bluish-black colour. The combination of the two symptoms has been found in one or two other instances, but there are clearly some cases of ochronosis in which there is no alkaptonuria.

Albumosuria.—A summary account of the recorded cases, and a discussion of its origin, is given by Parkes Weber [4] and colleagues. Multiple myeloma is held to include the entire class of leukæmias and pseudo-leukæmias, and to be due to an invasion by tumour cells from a growth in the leucocyte-forming tissues. In some of these diseases myelocyte cells are poured out, and the Bence Jones proteid is found in the urine. He concludes that this form of albumosuria is always due to a disease of bone marrow, affecting the myelocyte cells, and that it is usually accompanied with the formation of tumours from the unstable condition of these cells. Metastatic tumours in the bone marrow do not give rise to albumosuria, for their cells are not affected in the same way. J. H. Coriat, in the same journal, reports a case in which the Bence Jones proteid was found in large quantities in the pleuritic effusion of an insane patient suffering from multiple neuritis with extreme tenderness of the ribs. It was neither absorbed or passed into the blood-stream, as no trace of it appeared in the urine. He thinks it is produced from digestion of the proteids of the serum by the leucocytes or by bacteria, and that it is derived from serum globulin.

Albuminuria.—In place of the ordinary tests for albumen A. E. Wright [5] suggests finding the hæmolytic index, or the degree to which it must be diluted, to induce complete destruction of red cells in a standard solution. This gives a measure of the salts contained in the urine. Cryoscopy gives similar information, but the process is a somewhat intricate one. Gillespie [6] gives an abstract of Koranyi's work on the measurement of osmotic pressure by cryoscopy. Any substance in solution acts like a gas in a closed vessel, striving to occupy as large a space as possible, and so exerts osmotic pressure which varies with the temperature, concentration, and nature of the substance. The freezing point of a fluid solution is lowered in proportion to the number of molecules per unit of volume contained in it. Throughout the human organism there is a fairly constant osmotic pressure, and the kidneys have a regulative power by eliminating any excess of water or of molecules. Thus the metabolism of one molecule of albumen produces eighty-six of urea, hence the work placed upon the kidneys here is great. The freezing point of urine can only indicate the osmotic pressure in the blood if we take into account several factors. It varies in nephritis less than in health. The power of excreting water and molecules is lessened in parenchymatous nephritis, that of excreting solids is lessened in interstitial, and that of excreting water is lessened in chronic venous congestion. A low freezing point of urine is not always indicative of disease. In some cases of acute nephritis it may be very high. Copious ingestion of water in health lowers the freezing point far more than in (for instance) parenchymatous nephritis. The effects of saline solutions on the kidney are shown by some experiments of Castaigne and Rathery.[7] Kidney tissue was examined after half an hour's immersion in various strengths of sodium chloride solution. In solutions weaker than that whose freezing-point is ·78 C. the cells of the convoluted tubules are swollen. In strong solutions they are shrivelled. The straight tubules are not affected. In animals fed on salt free food albuminuria appears. If an excess of salt is given in health, no effect follows unless the excess is great or long continued. If the kidneys are diseased, the effects are serious and even fatal; intravenous saline injections in Bright's disease have caused fatal uræmia.

[1] Brit. Med. Jour., March 5. [2] Pract., March. [3] Lancet, Feb. [4] Amer. Jour. Med. Sci., Oct. [5] Lancet, April 2. [6] Edin. Med. Jour., April. [7] Med. Chron., Nov.

*(To be continued.)*

